# Pharmacokinetic/Pharmacodynamic Integration of Doxycycline Against *Mycoplasma hyopneumoniae* in an *In Vitro* Model

**DOI:** 10.3389/fphar.2019.01088

**Published:** 2019-09-20

**Authors:** Huilin Zhang, Chunxiao Mao, Jinju Li, Zilong Huang, Xiaoyan Gu, Xiangguang Shen, Huanzhong Ding

**Affiliations:** Guangdong Key Laboratory for Veterinary Drug Development and Safety Evaluation, South China Agricultural University, Guangzhou, China

**Keywords:** *Mycoplasma hyopneumoniae*, doxycycline, *in vitro* model, pharmacokinetic, pharmacodynamic

## Abstract

Doxycycline is a broad-spectrum antibacterial drug. It is used widely to treat diseases caused by *Mycoplasma* species. We investigated the antibacterial activity of doxycycline against the *Mycoplasma hyopneumoniae* strain ATCC25934. The minimum inhibitory concentration (MIC) of doxycycline against *M. hyopneumoniae* determined by a microdilution method was 0.125 μg/ml. Static time–kill curves with constant drug concentrations (0–64 MIC) showed that a bacteriostatic effect occurred if the doxycycline concentration reached 4 MIC. Doxycycline produced a maximum antimycoplasmal effect (reduction of 2.76 log_10_CFU/ml) at 64 MIC within 48 h. The effect of doxycycline against *M. hyopneumoniae* was analyzed by a sigmoid *E*_max_ model, and there was high correlation between the kill rate and doxycycline concentration (*R* ^2^ = 0.986). A one-compartment open model with first-order absorption was adopted and was used to simulate doxycycline pharmacokinetics in porcine plasma. The dynamic time–concentration curve showed that the area under the curve at 24 h (AUC_24 h_) and *C*_max_ (peak concentration) after each drug administration was 1.78–48.4 μg h/ml and 0.16–3.41 μg/ml, respectively. The reduction of *M. hyopneumoniae* (log_10_CFU/ml) for 1, 2.5, 5, 7.5, 10, 15, 20, and 30 mg/kg body weight was 0.16, 1.29, 1.75, 2.94, 3.35, 3.91, 4.35, and 5.77, respectively, during the entire experiment, respectively. When the dose was >10 mg/kg body weight, continuous administration for 3 days could achieve a bactericidal effect. The correlation coefficient of AUC_24 h_/MIC, *C*_max_/MIC, and %T > MIC (the cumulative percentage of time over a 24-h period that the drug concentration exceeds the MIC) with antibacterial effect was 0.917, 0.923, and 0.823, respectively. Doxycycline showed concentration-dependent activity, and the value of AUC_24 h_/MIC and *C*_max_/MIC required to produce a drop of 1 log_10_CFU/ml was 164 h and 9.89, respectively.

## Introduction

*Mycoplasma hyopneumoniae* is the primary etiologic agent of enzootic pneumonia and is widespread in pig populations (Rebaque et al., 2018). *M. hyopneumoniae* possesses a limited number of genes, low guanine and cytosine content and has simple metabolic pathways, so it cannot synthesize some essential compounds (Weber Sde et al., 2012). This microorganism is found primarily on the mucosal surface of the trachea, bronchi, and bronchioles. The immunity of pigs can be reduced significantly upon *M. hyopneumoniae* infection, which can induce infection by other pathogens. If *M. hyopneumoniae* is present with other pathogens, such as *Actinobacillus pleuropneumoniae*, *Pasteurella multocida*, *Streptococcus suis*, or *Haemophilus parasuis*, respiratory infections are aggravated and leads to porcine respiratory disease complex, which can cause considerable economic losses to the pig industry (Sibila et al., 2009).

As vaccine alone has limited antagonism to considerable variability of bacterial strain and has a lack of efficacy in stopping horizontal spread; therefore, antimicrobial chemotherapy is still necessary (Villarreal et al., 2011). *M. hyopneumoniae* is resistant to antimicrobial agents that interfere with the metabolism of folic acid and cell-wall synthesis, such as sulfonamides, trimethoprim, and the β-lactam class of antibiotics. Macrolides, tetracyclines, fluoroquinolones, lincosamides, and pleuromutilins are active agents against *M. hyopneumoniae* (Hannan et al., 1997; Maes et al., 2008).

Doxycycline is a tetracycline with good antimicrobial activity, strong penetration into tissue, and good oral bioavailability (Baert et al., 2000; De Mil et al., 2016; Li et al., 2016). Doxycycline binds to the decoding center of the small ribosomal subunit of the bacterium to inhibit protein synthesis. It is used for treatment of porcine respiratory diseases due to its broad-spectrum antibacterial activity. Doxycycline has been used widely for treatment of mycoplasmal pneumonia in pigs. There have been many studies on the pharmacokinetics (PK) of doxycycline not only in pigs but also in calves (Meijer et al., 1993), goats (Li et al., 2016), sheep (Castro et al., 2009; Castro Robles et al., 2012), broilers (Yang et al., 2018), cats (Hartmann et al., 2008), and dogs (Sebbag et al., 2018). The doxycycline concentration in pigs is best fitted to a one-compartmental model with first-order absorption (Bousquet et al., 1998).

Studies of treatment of pigs infected on a farm have indicated that doxycycline has significant therapeutic activity against mycoplasmal pneumonia (Wang et al., 2010; Liu, 2015). The nutritional and environmental conditions must be strict if *M. hyopneumoniae* is cultured *in vitro*, and isolating clinical strains is difficult. Limited information is available on the PK/pharmacodynamic (PD) interactions of antibacterial agents against *Mycoplasma* species, including doxycycline. However, in recent years, development of an *in vitro* PK/PD model has produced a robust method for correlation research (de la Pena et al., 2004; Budha et al., 2009; Nicasio et al., 2012). Mitchell et al. (2013) studied the antimicrobial activity of various agents against *Mycoplasma mycoides* subspecies *mycoides* small colony (MmmSC) *in vitro*. The dilution model they used was simulated by increasing the volume, which cannot mimic the continuous process of drug elimination. Zhang et al. (2016) evaluated antimicrobial activity against *Mycoplasma gallisepticum* using a simple *in vitro* dynamic model that could eliminate the agent through an outlet, and the initial antimicrobial concentrations corresponded to various multiples of the minimum inhibitory concentration (MIC).

We investigated the effect of doxycycline against *M. hyopneumoniae* based on PK/PD interactions. The model was based on the studies mentioned above with some necessary modifications: (i) we added an absorption chamber to mimic absorption in animals, and (ii) the design of the dose regimen was in accordance with clinical practice. We wanted to provide a reference for dose-regimen optimization which could achieve a cure for *Mycoplasma pneumoniae* infection and minimize the opportunity for antimicrobial resistance.

We pursued four objectives: (i) the MIC of doxycycline against *M. hyopneumoniae* strain ATCC25934 was determined; (ii) the *in vitro* time–kill curves for doxycycline against *M. hyopneumoniae* in a defined artificial medium were established and the relationship between the kill rate and drug concentrations were fitted to the *E*_max_ model (see below); (iii) the relationship between PK/PD indices and the effect of doxycycline against *M. hyopneumoniae* were investigated in an *in vitro* dynamic PK/PD model; and (iv) a scientific dosing guidance of doxycycline against *M. hyopneumoniae* was provided based on PK/PD modeling.

## Materials and Methods

### Materials

The standard strain of *M. hyopneumoniae* (ATCC25934) was provided by the Chinese Veterinary Microorganism Culture Collection Center (Beijing, China). A doxycycline hyclate standard (>99%) was obtained from the China Institute of Veterinary Drug Control (Beijing, China). The artificial medium base of *M. hyopneumoniae* was purchased from Qingdao Hope Biological Technology (Qingdao, China). Sterile pig serum was supplied by Guangzhou Ruite Biological Technology (Guangzhou, China). Cysteine and the reduced form of nicotinamide adenine dinucleotide were supplied by Guangzhou Prob Information Technology. The “raw” form of doxycycline (87.4%) was provided by Guangdong Dahuanong Animal Health Products (Guangdong, China).

### MIC Determination

A microdilution method was used to determine the MIC of doxycycline against *M. hyopneumoniae* strain ATCC25934, as described by Tanner et al. (1993) with modifications. The exponential-phase culture was diluted with culture medium to the desired inoculum size of 10^6^ CFU/ml. The culture medium of *M. hyopneumoniae* contained artificial medium base, 20% pig serum, trace cysteine, and nicotinamide adenine dinucleotide. A series of concentrations of doxycycline was prepared by twofold dilution (final concentration was 0.001–0.25 μg/ml) with media. Each of the first to the ninth column was added 0.1 ml of media with different concentrations of doxycycline and 0.1 ml of *M. hyopneumoniae* inoculum. A growth control (*M. hyopneumoniae* inoculum in the absence of antimicrobial agents), an end-point control (blank medium at pH 6.8) and a sterility control (sterility medium) were included. After sealing with a gas-permeable film, plates were cultured at 37°C in an atmosphere of 5% CO_2_. The MIC was determined as the minimum concentration of doxycycline that resulted in no change in color. All experiments were carried out in triplicate.

### Exposure to Static Antibiotic Concentrations

We determined the effect of doxycycline against *M. hyopneumoniae* at static drug concentrations. The doxycycline concentrations used were 0, 0.5, 1, 2, 4, 6, 8, 16, 32, and 64 MIC. Then, 0.4 ml of the *M. hyopneumoniae* suspension in the exponential phase, 0.1 ml of drug solution, and 3.5 ml of culture medium were added to a 7-ml penicillin bottle. The initial inoculum size was ∼10^6^ CFU/ml. A growth control (*M. hyopneumoniae* culture without antimicrobial agents) and sterility control (4 ml of blank medium) were included. Cultures were incubated at 37°C in an atmosphere of 5% CO_2_ for 48 h, and 100 μl of each culture was collected at 0, 1, 3, 6, 9, 12, 24, 36, and 48 h for *M. hyopneumoniae* counting. Each sample was serially diluted 10-fold, then 10-µl dilutions were transferred to agar plates. The latter were incubated for 7 days at 37°C in a humidified incubator in an atmosphere of 5% CO_2_. Then, the number of *M. hyopneumoniae* colonies was recorded using an inverted microscope (Leica, Germany). The limit of detection was 100 CFU/ml. Time–kill studies were repeated at least thrice on different days.

### Static Time–Kill Curves Fitting and Analysis

The kill rate can reflect the effect of antibacterial drugs against a pathogen. The greater the growth rate, the stronger the antibacterial effect of the drug. The time–kill curves of doxycycline against *M. hyopneumoniae* were presented by plotting log_10_CFU/ml against time (h) at different concentrations. First, the kill rates of 0–24 h, 0–36 h, and 0–48 h time periods were calculated. The kill rates of 3–24 h, 3–36 h, and 3–48 h were also calculated because that a recovery growth was observed after transferring to the new culture vessel. Besides, the kill rates were also calculated from 12 and 48 h because there was no significant reduction in *M. hyopneumoniae* counts within 12 h. Thus, eventually, the mean kill rate for seven time intervals (0–24 h, 0–36 h, 0–48 h, 3–24 h, 3–36 h, 3–48 h, and 12–48 h) was calculated by linear regression. The mean kill rate for these seven time intervals was selected to fit the drug concentration using the *E*_max_ model employing WinNonlin 6.1 (Pharsight, Mountain View, Sunnyvale, CA, USA). The *E*_max_ model can be presented as follows:

$$E=\frac{E_{\max}\times C_{e}^{N}}{{\mathrm{EC}}_{50}^{N}+C_{e}^{N}}$$

where *E* is the kill rate, *E*_max_ is the maximum value of kill rate in a certain time interval, *C*_e_ is the doxycycline concentration, *N* is the Hill coefficient (which describes the steepness of the kill rate–concentration curve), EC_50_ is the concentration at which 50% of the maximum kill rate is reached, and *R* ^2^ is the correlation coefficient of the relationship between experimental values and predicted values.

### Description of the *In Vitro* Dynamic Model

According to the *in vitro* dynamic model previously established in our laboratory (Zhang et al., 2016; Zhang et al., 2018b; Huang et al., 2019). The study adopted a one-compartment open model with first-order absorption established by Huang et al. (2019) (**Figure 2**). This model was used to simulate the PK of doxycycline in porcine plasma (absorption half-life of 4.15 h, elimination time of 5.9 h) (Bousquet et al., 1998). The model contained four compartments: an absorption chamber, a central chamber, a reserve chamber, and a recycle chamber. The model is based on an absorption chamber to simulate absorption and a central chamber in which the drug works. The absorption chamber acted as administration site, which contained 210 ml sterile medium. The central chamber comprised 300 ml of sterile medium (external compartment) and a 10-ml volume dialysis tube (internal compartment) to culture bacteria. A reserve chamber was used to provide fresh medium, and an empty vessel was employed to collect waste. The compartments were connected by a fiberglass tube. Peristaltic pumps were used to operate the model, and the flowrate of the peristaltic pumps was set at 0.58 ml/min. Stirring hot plates were employed to mix the drug and provide a growth temperature (37°C) for *M. hyopneumoniae*.

### Exposure to Dynamic Antibiotic Concentrations

Ten milliliters of inoculum of turbidity 10^7^ CFU/ml was administered to the central dialysis tube. After *M. hyopneumoniae* had adapted to the new growth environment, and it was determined that the entire device was not contaminated; a certain drug dose was administered to the absorption chamber: this time was regarded as the initial time of the experiment. According to the clinically recommended dose, eight dose groups (1, 2.5, 5, 7.5, 10, 15, 20, and 30 mg/kg body weight) were designed in the *in vitro* dynamic model once daily for 3 days. Sampling was done 0 h before administration as well as 1, 2, 4, 6, 12, 24, 30, 36, 48, 54, 60, 72, 84, and 96 h after administration for determination of the drug concentration). in addition, 0.1 ml of inoculum in the dialysis tube was sampled 0 h before administration as well as 6, 12, 24, 30, 36, 48, 54, 60, 72, 84, and 96 h after administration for bacterial counting. All experiments were carried out in triplicate.

### Susceptibility Testing of *M. hyopneumoniae*

The samples were collected at 24, 48, 72, and 96 h from a dialysis tube, which were cultured to the logarithmic growth phase. Then, the exponential-phase culture was diluted to the inoculum size of 10^6^ CFU/ml, and 10 μl of dilution was dropped on the surface of drug plates that contained 1 MIC concentration. After 7 days of culture, the colonies recovered on the plate were inoculated into the liquid medium for further culture. After continuous passaging for five times, the MIC of the strain was determined by a microdilution method. If the MIC value of the strain remained high, it indicates that the strain reduced sensitivity.

### Intergration and Modeling of PK/PD

PK/PD parameters were derived from time–concentration assays, and the antimycoplasmal effect was obtained from dynamic time–kill curves. The area under the concentration–time curve at 24 h (AUC_24 h_) was calculated using the trapezoidal method, and the *C*_max_ after each drug administration was observed directly from the curve. The parameter of %T > MIC were calculated according to a noncompartmental analysis by WinNonlin. Then, data were analyzed using the sigmoid *E*_max_ model as follows:

$$E=E_{\max}-\frac{(E_{\max}-E_{0})\times C_{e}^{N}}{{\mathrm{EC}}_{50}^{N}+C_{e}^{N}}$$

where *E* is the antimycoplasmal effect; *E*_max_ is the change of control sample (log_10_CFU/ml) in periods of 0–24 h after each administration; *E*_0_ is the maximum value of the antimycoplasmal effect; *C*_e_ is the value of PK/PD indices (*C*_max_/MIC, AUC_24 h_/MIC and %T > MIC); EC_50_ is the PK/PD index that produces a 50% reduction of the maximum antimycoplasmal effect; and *N* is the Hill coefficient.

## Results

### Minimum Inhibitory Concentration

The MIC of doxycycline against *M. hyopneumoniae* ATCC25934 as determined by the microdilution method was 0.125 μg/ml.

### Static Time–Kill Curves and Analysis

**Figure 1:**
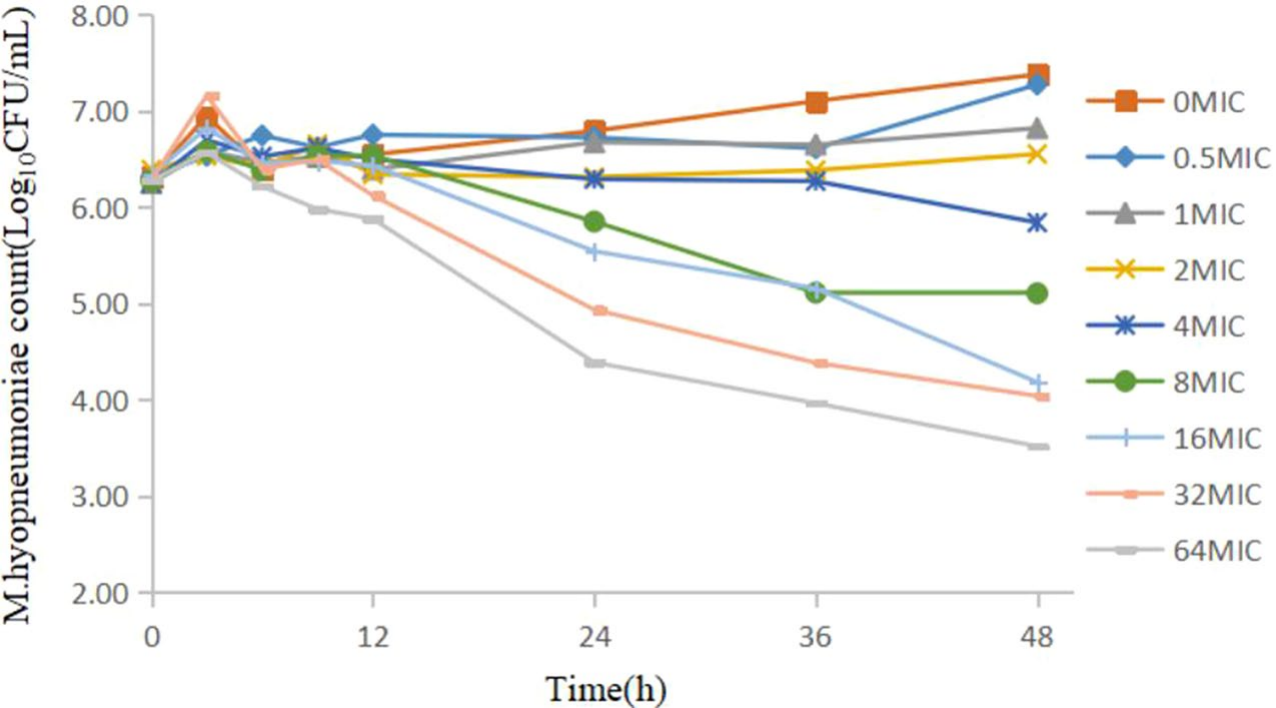
Static time–kill curve of doxycycline against *Mycoplasma hyopneumoniae* at different concentrations.

The static time–kill curve is shown in **Figure 1**: doxycycline produced a marked reduction in the range 0.41–2.76 log_10_CFU/ml within 48 h. When the drug concentration was <2 MIC, mild inhibition of *M. hyopneumoniae* proliferation compared with that in the growth control group was observed, and *M. hyopneumoniae* showed a slow increase relative to the initial inoculum size. When the drug concentration was >4 MIC, doxycycline showed bacteriostatic activity against *M. hyopneumoniae* within 48 h but did not show a bactericidal effect at all doses. There was no obvious change in the number of *M. hyopneumoniae* colonies within 12 h, except for a decrease at <64 MIC, which had a reduction of 0.39 log_10_CFU/ml. **Figure 1** shows that a higher concentration of antibiotic elicited a better antibacterial effect.

**Figure 2:**
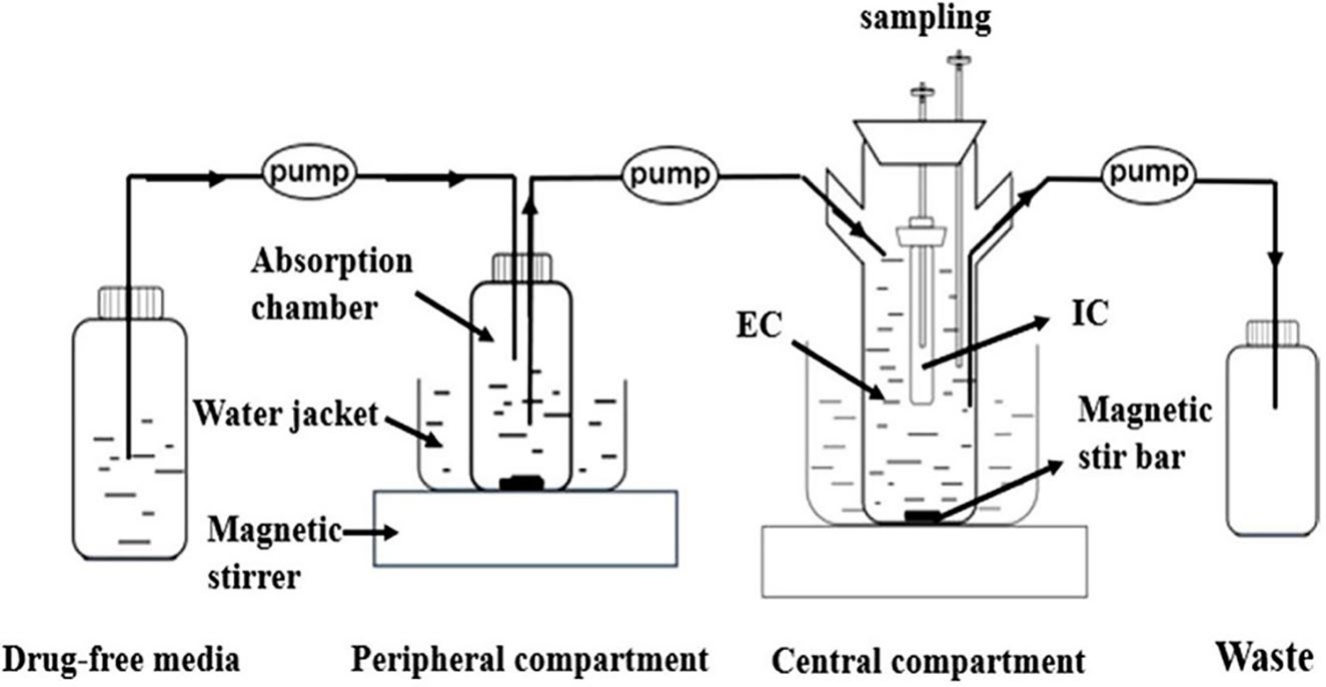
The *in vitro* model that simulates the pharmacokinetics of doxycycline and determines a drug’s effect on the growth and susceptibility of *M. hyopneumoniae*.

The relationship between the kill rate and concentration is presented in **Figure 2** (time interval was 12–48 h). The *R* ^2^ estimated from the different time intervals ranged from 0.970 to 0.986. The maximum *R* ^2^ (0.986) arose during the period 12–48 h, and the maximum kill rate was 0.11/h. The profile of the kill rate demonstrated that, if the concentration was ≤16 MIC, the kill rate increased with increasing concentration. The relationship between concentration and kill rate was fitted by the *E*_max_ model, and the obtained parameters of *E*_max_, EC_50_, and the Hill coefficient are presented in **Table 1**.

**Table 1:** The kill rate parameter estimation derived from the *E*_max_ model.

Time (h)	*E* *_max_* (h^−1^)	EC_50_ (μg/ml)	Hill’s slope	***R*** ***^2^***
0–24	0.09	2.82	1.73	0.974
0–36	0.07	1.62	1.75	0.970
0–48	0.05	1.09	2.53	0.975
3–24	0.24	13.04	0.78	0.947
3–36	0.09	1.18	1.74	0.985
3–48	0.07	0.98	2.2	0.985
12–48	0.06	0.78	2.98	0.986

E_max_, the maximum value of kill rate in a certain time interval; EC_50_, the concentration at which 50% of the maximum kill rate is reached; R^2^, the correlation coefficient of the relationship between experimental values and predicted values.

### Pharmacokinetics in the *In Vitro* Dynamic Model

**Figure 3:**
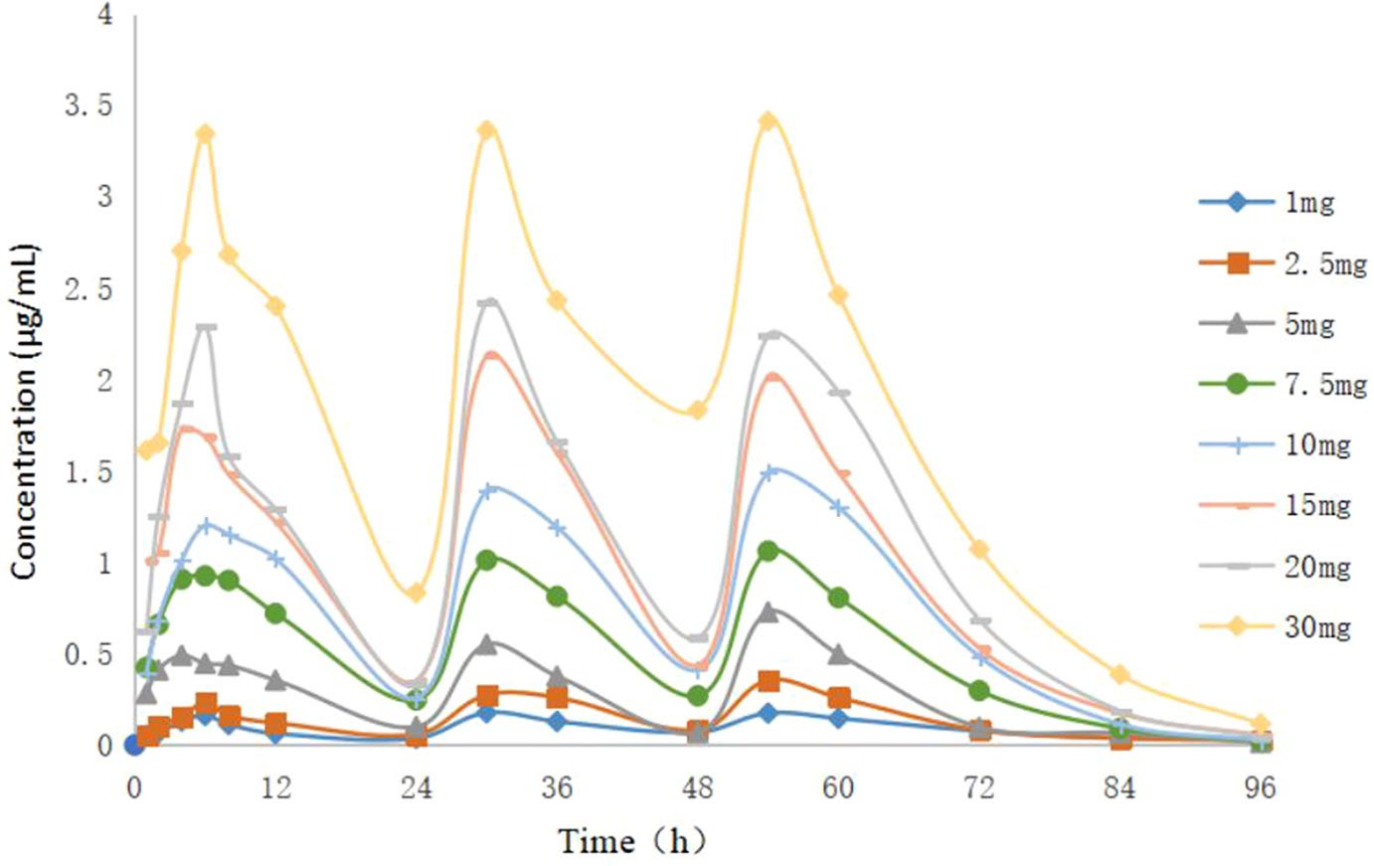
Concentration–time curve of doxycycline in the dynamic mode.

**Figure 4:**
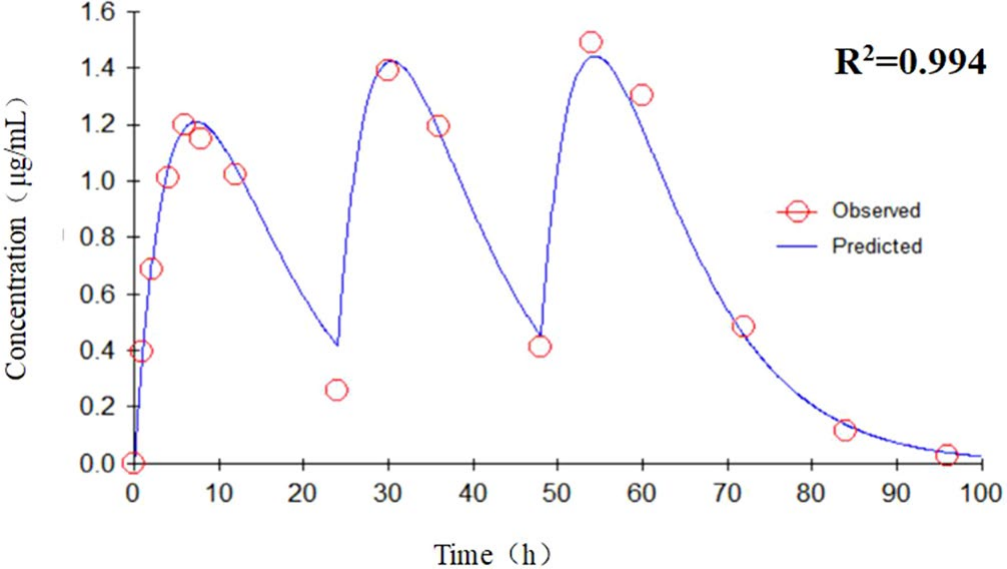
The concentration–time profile fitted to a one-compartment open model with first-order absorption by WinNonlin.

The concentration–time curve of the *in vitro* dynamic model for doxycycline is displayed in **Figure 3**: the peak concentration was reached ∼6 h after each doxycycline administration. AUC_24 h_ and *C*_max_ after each drug administration was 1.78–48.4 μg h/ml and 0.16–3.41 μg/ml, respectively. According to the recommended dose of 10 mg/kg body weight, the peak concentration could reach 1.49 μg/ml in a single administration, which is well above the MIC (0.125 μg/ml) of doxycycline against *M. hyopneumoniae*. Data were analyzed according to a one-compartmental model by WinNonlin and are shown in **Figure 4**. The concentration–time profile of doxycycline in the *in vitro* dynamic model fitted a one-compartment open model with first-order absorption (*R* ^2^ = 0.994).

### Pharmacokinetic/Pharmacodynamic Modeling and Analysis

**Figure 5:**
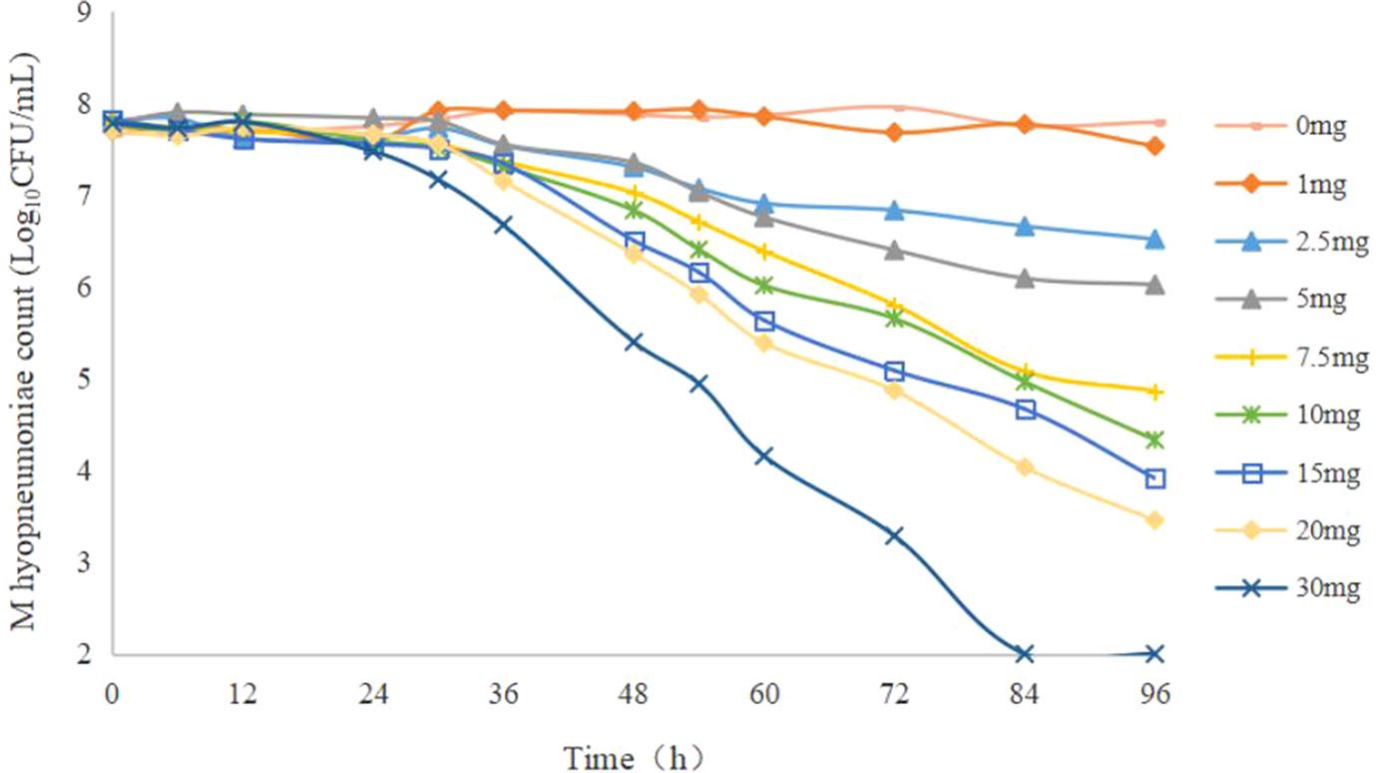
Dynamic time–kill curve at different simulated doses.

**Figure 5** shows the antibacterial efficacy of doxycycline against *M. hyopneumoniae* at eight simulated dosing regimens. The reduction in bacterium (log_10_CFU/ml) for several dosing regimens (1, 2.5, 5, 7.5, 10, 15, 20, and 30 mg/kg body weight) was 0.16, 1.29, 1.75, 2.94, 3.35, 3.91, 4.35, and 5.77 during the entire experiment, respectively. Within the interaction at 24 h, there was no marked decrease in the maximum reduction (0.31 log_10_CFU/ml). When the dose was <7.5 mg/kg body weight, doxycycline could kill *M. hyopneumoniae* (maximum reduction = 2.94 log_10_CFU/ml). When the dose was >10 mg/kg body weight, continuous administration for 3 days could achieve a bactericidal effect. At 30 mg/kg body weight, the number of *M. hyopneumoniae* could fall below the limit of detection (100 CFU/ml) during the entire experiment. Hence, doxycycline had a significant effect on *M. hyopneumoniae*, and escalating doses of doxycycline resulted in marked killing of *M. hyopneumoniae*. There have no drug-resistant strains been obtained under various dosing regimens.

**Figure 6:**
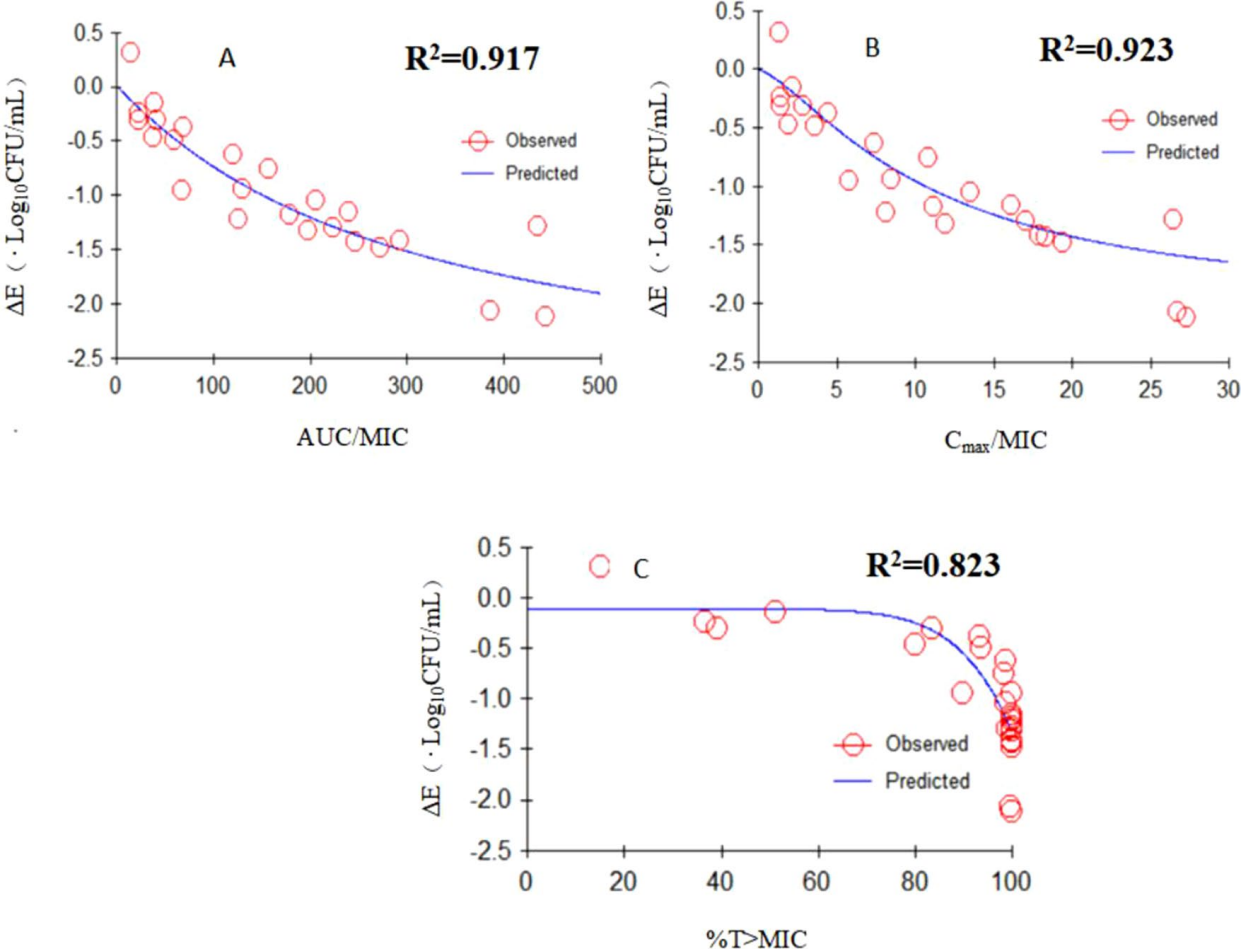
*E*_max_ relationships for the three PK/PD parameters versus antimycoplasmal effect. **(A)** AUC_24 h _/MIC; **(B)** *C*_max_/MIC; **(C)** *T* > MIC.

The *E*_max_ relationships of three PK/PD parameters (AUC_24 h_/MIC, *C*_max_/MIC, T > MIC) versus the antimycoplasmal effect are shown in **Figure 6**. The *R* ^2^ of AUC_24 h_/MIC, *C*_max_/MIC, and %T > MIC with an antimycoplasmal effect was 0.917, 0.923, and 0.823, respectively. These results suggested that the effect of doxycycline against *M. hyopneumoniae* was concentration dependent and that efficacy was driven by AUC_24 h_/MIC and *C*_max_/MIC. The relationship between antibacterial efficacy and PK/PD parameters was assessed by a sigmoid *E*_max_ model, and the obtained parameters of *E*_0_, *E*_max_, and EC_50_, and Hill coefficient are presented in **Table 2**. The AUC_24 h_/MIC and *C*_max_/MIC for producing a drop in log_10_CFU/ml of 1 was 164 h and 9.89, respectively.

**Table 2:** The kill rate parameter estimation derived from the *E*_max_ model.

PK/PD parameter	AUC_24 h_/MIC (h)	*C*_max_/MIC
*E*_max_ (log_10_CFU/ml)	−3×10^−05^	−5×10^−05^
EC_50_	179	21
*E*_0_ (log_10_CFU/ml)	−2.13	−3.14
Hill’s slope	1.38	1.01
*R* ^2^	0.917	0.923
1 log_10_CFU/ml drop	164 h	9.89

AUC_24h_/MIC, the area under the curve at 24 h by MIC; *C*_max_/MIC, peak concentration by MIC; *E*_max_, the change of control sample (log_10_CFU/ml) in periods of 0–24 h after each administration; *E*_0_, the maximum value of the antimycoplasmal effect; EC_50_, the PK/PD index that produces a 50% reduction of the maximum antimycoplasmal effect.

## Discussion

*In vitro* dynamic models provide conditions for the studying of PK/PD of *mycoplasma*. *M. hyopneumoniae* is an important infectious pathogen in pigs and causes major economic losses to the pig industry. However, relevant studies have been hindered by three main difficulties. First, the characteristics of *Mycoplasma* species (small genome, small volume, and absence of a cell wall) mean that the nutrients and environment for *in vitro* culture must be controlled strictly (Beier and Siqueira, 2018). Some *Mycoplasma* strains can be cultured in a rich and complex medium supplemented with a large amount of serum, but the turbidity in media limits research. Second, isolating *M. hyopneumoniae* is very difficult because of its slow growth (and potential overgrowth) with other *Mycoplasma* species (Maes et al., 2018). Third, *M. hyopneumoniae* infection is complicated, lengthy, and affected by strain diversity, virulence, and host autoimmunity, which makes establishment of animal models difficult (Razin and Jacobs, 1992). Limited information is available on the PK/PD interactions of antibacterial agents against *Mycoplasma* species using *in vitro* or animal PK/PD models. In the present study, a one-compartment open model with first-order absorption was adopted and was used to simulate the PK of doxycycline in porcine plasma.

This PK/PD model reflects the relationship between the drug, bacteria, and quantification of the activity (as well as the likely efficacy) of antimicrobial agents against target pathogens (Zhang et al., 2018a). The dynamic model that we used provided a continuous flow of doxycycline to simulate the PK of doxycycline in pigs. In addition, a dialysis tube was used to culture bacteria, which (i) provided a steady source of nutrients for *M. hyopneumoniae* and (ii) avoided loss of *M. hyopneumoniae*. Samples could be collected continuously to monitor the growth and death of *M. hyopneumoniae*.

The PK/PD model used in the study is functionally superior to other models. Compared with the older dynamic model using only the central effect chamber, this model adds an absorption chamber to simulate drug absorption. Bacteria were cultured in dialysis tubes that allowed exchange of drug and nutrients (Meletiadis et al., 2012). We counted *M. hyopneumoniae* colonies on agar plates to investigate the effect of doxycycline against *M. hyopneumoniae.* The *in vitro* dynamic time–kill curve could characterize the activity of doxycycline against *M. hyopneumoniae* preliminarily. Where appropriate, data were fitted to the sigmoid *E*_max_ model to obtain specific PK/PD parameters, and the PK/PD index obtained was used to calculate the parameter value corresponding to a certain antibacterial effect. We could investigate the PK/PD interactions of doxycycline against *M. hyopneumoniae* to determine the concentration and/or time dependence of such activity and provide guidance for designing dose regimens for the clinical treatment of *M. hyopneumoniae* infection.

Referring to relevant study reports, the value of MIC is within reasonable range. The MIC for doxycycline against *M. hyopneumoniae* determined using a microdilution method was 0.125 μg/ml. To be consistent with the *in vitro* experiment of time–kill curves, the final inoculum of *M. hyopneumoniae* in each well was 10^6^ CFU/ml in our study. The recommended turbidity of MIC testing against veterinary *Mycoplasma* species is 10^3^–10^5^ CFU/ml (Hannan, 2000). The turbidity of the growth phase of *M. hyopneumoniae* seems to be not so important, and the inoculum size exhibits little effect on the MIC determination of *Mycoplasma* species. The test strain ATCC25934 is a standard strain of *M. hyopneumoniae*, and its antibiotic sensitivity is different in different clinical isolates to some extent. Felde et al. (2018) calculated the MIC value of doxycycline against 44 *M. hyopneumoniae* field strains isolated from Central Europe by a microbroth-dilution method. MIC_50_ (the minimal concentration that inhibits colony formation by 50%) and MIC_90_ (the minimal concentration that inhibits colony formation by 90%) was 0.078 and 0.312 μg/ml, respectively. Moreover, most studies from Europe have demonstrated similar values of MIC_50_ and MIC_90_ for oxytetracycline (Tavio et al., 2014).

The study has shown that doxycycline has a significant effect on *M. hyopneumoniae*. **Figure 1** shows the static time–kill curve. When the doxycycline concentration was >4 MIC, doxycycline showed bacteriostatic activity against *M. hyopneumoniae* within 48 h and produced a maximum reduction of 2.76 log_10_CFU/ml at 64 MIC. **Figure 5** shows the antibacterial efficacy of doxycycline against *M. hyopneumoniae* in our dynamic PK/PD model. We found that doxycycline could kill *M. hyopneumoniae*. When the dose was >10 mg/kg body weight, continuous interactions could result in a bactericidal effect during the experiment. At the dose of 30 mg/kg body weight, doxycycline produced a maximum antimycoplasmal effect of 5.77 log_10_CFU/ml reduction within 96 h. **Figure 5** indicated that escalating doses of doxycycline resulted in marked killing of *M. hyopneumoniae*. The results of *E*_max_ model verified that the antibacterial effect of doxycycline was concentration dependent, in which the relationship between antimycoplasmal effect and PK/PD parameters was fitted. The magnitude of AUC_24 h_/MIC and *C*_max_/MIC predicted for 1 log_10_ (CFU/ml) reduction was 164 h and 9.89, respectively. These data provided a scientific dosing guidance of doxycycline against *M. hyopneumoniae*.

There have no drug-resistant strains been obtained in this study. We tried to screen for drug-resistant strains of *M. hyopneumoniae* in an *in vitro* dynamic model using drug-containing agar plates. No drug-resistant strains were obtained under various dosing regimens during short-term experimental studies. The result indicated that *M. hyopneumoniae* is not easy to get resistant for doxycycline.

Studies have shown that doxycycline does not have activity against MmmSC if the bacteria are exposed to the drug for 12 and 24 h at a constant concentration (Mitchell et al., 2013). Those findings are not in accordance with a study that indicated doxycycline had a marked effect on *M. gallisepticum* (Zhang et al., 2016). In this study, irrespective of whether a static or dynamic drug concentration was used, there was no significant reduction in *M. hyopneumoniae* counts within several hours of the start of the experiment. There could be three explanations for these results. First, delayed growth of *M. hyopneumoniae* was observed, and *M. hyopneumoniae* did not recover instantaneously in the logarithmic growth phase upon transfer to the new culture environment. Second, the antibacterial mechanism of doxycycline is to inhibit protein synthesis, and it acted as a long-acting bacteriostatic agent (Schmidt et al., 2018; Chukwudi and Good, 2019). Third, due to the slow growth of *M. hyopneumoniae*, bacteriostatic agents (e.g., doxycycline) need longer to elicit their effects.

According to the bactericidal properties of drug on the bacteria, drugs can be divided into time- and concentration-dependent drugs. When the antibiotic concentration is increased, concentration-dependent drugs lead to faster killing, whereas the kill rate of time-dependent drugs might be remained constant (Craig, 1998). In this study, static time-killing and dynamic study both showed that a higher concentration of antibiotic elicited a better antibacterial effect. The results of dynamic time-killing study showed that the *C*_max_/MIC parameter had the highest correlation with the antibacterial effects (*R* ^2^ = 0.923). Therefore, we could infer that the antimicrobial effect of doxycycline against *M. hyopneumoniae* was concentration dependent. In this study, WinNonlin was employed to fit the drug concentration and kill rate (obtained from different intervals). The results suggest that higher doxycycline concentration result in faster kill rate until the doxycycline concentration was >16 MIC. In traditional PK/PD studies, bacteria are exposed to a constant drug concentration, and efficacy evaluation is based on time–kill curves (Zhanel et al., 1991). This method can be used to study the initial kill rate of bacteria and the ability of bacterial regrowth. However, the drug concentrations in serum and target tissue are changing continuously, so the dynamic model used in our study is clinically important. The rapid killing observed in the *in vitro* dynamic time–kill curves suggested that doxycycline exhibited a concentration-dependent killing mechanism against *M. hyopneumoniae*. Data were fitted to a sigmoidal *E*_max_ model, and the PK/PD parameters that correlated most closely to efficacy were AUC_24 h_/MIC (*R* ^2^ = 0.917) and *C*_max_/MIC (*R* ^2^ = 0.923). Doxycycline seemed to be driven by the AUC.

However, this result is not in complete accordance with the literatures that have been made about the PDs of doxycycline. The effect of doxycycline on Gram-positive pathogens (e.g., *Staphyloccus aureus*, *Steptococcus pneumonia*) and Gram-negative pathogens (e.g., *Pasteurella multocida*, *Escherichia coli*) suggests that doxycycline kills bacteria by time-dependent kinetics at low concentrations but by concentration-dependent kinetics at high concentrations (Cunha et al., 2000). In a study of the effects of doxycycline against *M. gallisepticum*, the time–kill curves showed time-dependent characteristics (Zhang et al., 2016). Hence, at present, this area is understood incompletely, and our study will aid understanding (Agwuh and MacGowan, 2006). In addition, the concentration-dependent characteristics must be confirmed by further studies using an *in vivo* PK/PD model and other *Mycoplasma* strains.

## Data Availability

The datasets generated for this study are available on request to the corresponding author.

## Author Contributions

HD and HZ conceived and designed the experiments. HZ, CM, and JL performed the experiments. HZ and ZH analyzed data and wrote the manuscript. XG, XS, and HD revised the manuscript and supervision of the entire project. All authors read and approved the final manuscript.

## Funding

This work was supported by the National Key Research and Development Program of China (grant numbers 2016YFD0501300 and 2016YFD0501310).

## Conflict of Interest Statement

The authors declare that the research was conducted in the absence of any commercial or financial relationships that could be construed as a potential conflict of interest.
